# Pressure-Driven Polar Orthorhombic to Tetragonal Phase Transition in Hafnia at Room Temperature

**DOI:** 10.1021/acs.chemmater.4c02596

**Published:** 2025-02-17

**Authors:** Janice L. Musfeldt, Sobhit Singh, Kevin A. Smith, Xianghan Xu, Sang-Wook Cheong, Zhenxian Liu, David Vanderbilt, Karin M. Rabe

**Affiliations:** † Department of Chemistry, University of Tennessee, Knoxville, Tennessee 37996, United States; ‡ Department of Physics and Astronomy, University of Tennessee, Knoxville, Tennessee 37996, United States; § Department of Mechanical Engineering, University of Rochester, Rochester, New York 14627, United States; ∥ Materials Science Program, University of Rochester, Rochester, New York 14627, United States; ⊥ Department of Physics and Astronomy, Rutgers University, Piscataway, New Jersey 08854, United States; # Rutgers Center for Emergent Materials, Rutgers University, Piscataway, New Jersey 08854, United States; ∇ Department of Physics, University of Illinois Chicago, Chicago, Illinois 60607-7059, United States

## Abstract

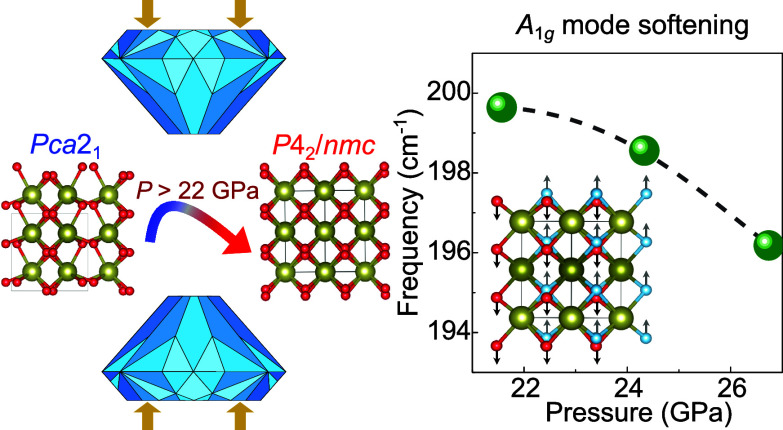

Oxides are legendary for their complex energy landscapes, sensitivity to external stimuli, and property control through chemical substitution. Of these, the binary oxide HfO_2_ is one of the most fascinating because of the extraordinary number competing phases and opportunities to stabilize unique and useful properties. In this work, we combined synchrotron-based infrared absorbance and Raman scattering spectroscopies with diamond anvil cell techniques and first-principles calculations to explore the properties of polar orthorhombic hafnia (chemical formula HfO_2_:*x*Y, where *x* = 12%) under pressure. Compression drives this system to the tetragonal form above 22 GPa—quite different from the more conventional phase diagram derived from pressurization of monoclinic HfO_2_ where the tetragonal phase resides at elevated temperatures. In addition to evidence for a complex energy landscape, we unveil a wide coexistence region, order-of-magnitude differences in phonon lifetimes, and an *A*_1*g*_ symmetry phonon in the tetragonal phase with a negative mode Grüneisen parameter that drives the system toward the cubic phase. Similar pressure pathways may connect other metastable phases in this family of materials.

## Introduction

Since 2007, amorphous hafnia (HfO_2_) thin films have been used as high κ gate dielectrics in CMOS technology, enabling the continuation of Moore’s scaling of DRAM chips.^1−3^ The discovery of ferroelectricity in thin films^4^ which, unlike that of conventional perovskite ferroelectrics,^5^ is robust at thicknesses down to the unit-cell scale^6^ and is highly compatible with silicon^7−9^ makes hafnia an attractive candidate for nonvolatile ferroelectric FET devices^8,9^ and negative capacitance heterostructures.^10,11^ Further exploration of HfO_2_ has revealed a rich energy landscape of competing phases in crystalline thin films of hafnia,^12−14^ including phases with planar polar domains whose polarization directions can be chosen almost independently,^6^ suggesting yet more state-of-the-art device applications.^15,16^

Various approaches have been advanced to explain the origin of multiple competing phases and the ferroelectric phase in particular.^12,13,17−23^ It is natural to use the *Fm*3̅*m* cubic fluorite phase as a reference state. This phase has a single lattice instability that generates the centrosymmetric *P*4_2_/*nmc* tetragonal phase as a local energy minimum; this from which the orthorhombic ferroelectric phase can be obtained through an improper trilinear coupling mechanism, involves a combination of polar, nonpolar, and antipolar phonon modes.^12,13,17−20^ On the other hand, the use of other high-symmetry reference structures, for example an antipolar orthorhombic *Pbcn* structure, gives an alternative route to the orthorhombic phase through a polar instability, reminiscent of proper ferroelectrics.^24−26^ In this reference structure, there are several flat branches of unstable phonons, which naturally give rise to the phases with planar polar domains with widths on the unit cell scale. A metastable tetragonal phase (*P*4_2_/*nmc*), corresponding to the ambient pressure tetragonal phase observed at temperatures above 1720 °C and at slightly lower temperatures under pressure,^27^ is predicted to take part in various structural transformation and ferroelectric switching pathways,^18,24,25^ but it has remained elusive in single crystal form at 300 K (although it appears at high temperatures when monoclinic hafnia is compressed). A detailed investigation of phonons and the distortion pathways that they represent^28−31^ is therefore key to identifying and connecting the competing metastable states that involve tetragonal hafnia. More generally, it may be possible to access interesting metastable phases by driving the system along appropriate pressure pathways through the complex energy landscape that involve other metastable states.

The growth of high quality HfO_2_:*x*%Y single crystals (*x* = 0, 7, 11, 12, 20%)^32^ is a breakthrough that has created a number of unprecedented opportunities to explore structure–property relations in this family of materials.^32−35^ While at room temperature the undoped state is monoclinic (*P*2_1_/*c*), different Y concentrations support the antipolar orthorhombic phase (*Pbca*), the polar orthorhombic phase (*Pca*2_1_), and the high-energy cubic form (*Fm*3̅*m*).^32^ The *Pca*2_1_ polar orthorhombic phase of HfO_2_:12%Y hosts switchable ferroelectricity with a magnitude of 3 μC/cm^2^ and a coercive electric field of 4 MV/cm.^32^ Evidence for this state comes from X-ray, TEM, and vibrational spectroscopies.^29,32^ The antipolar *Pbca* phase is stabilized in HfO_2_:11%Y and can be prepared by mixed phase materials via pressure cycling.^36^ The antiferroelectric orthorhombic material has energy applications^37^ and is implicated in the switching pathway of the polar orthorhombic material.^19,24^

**Figure 1:**
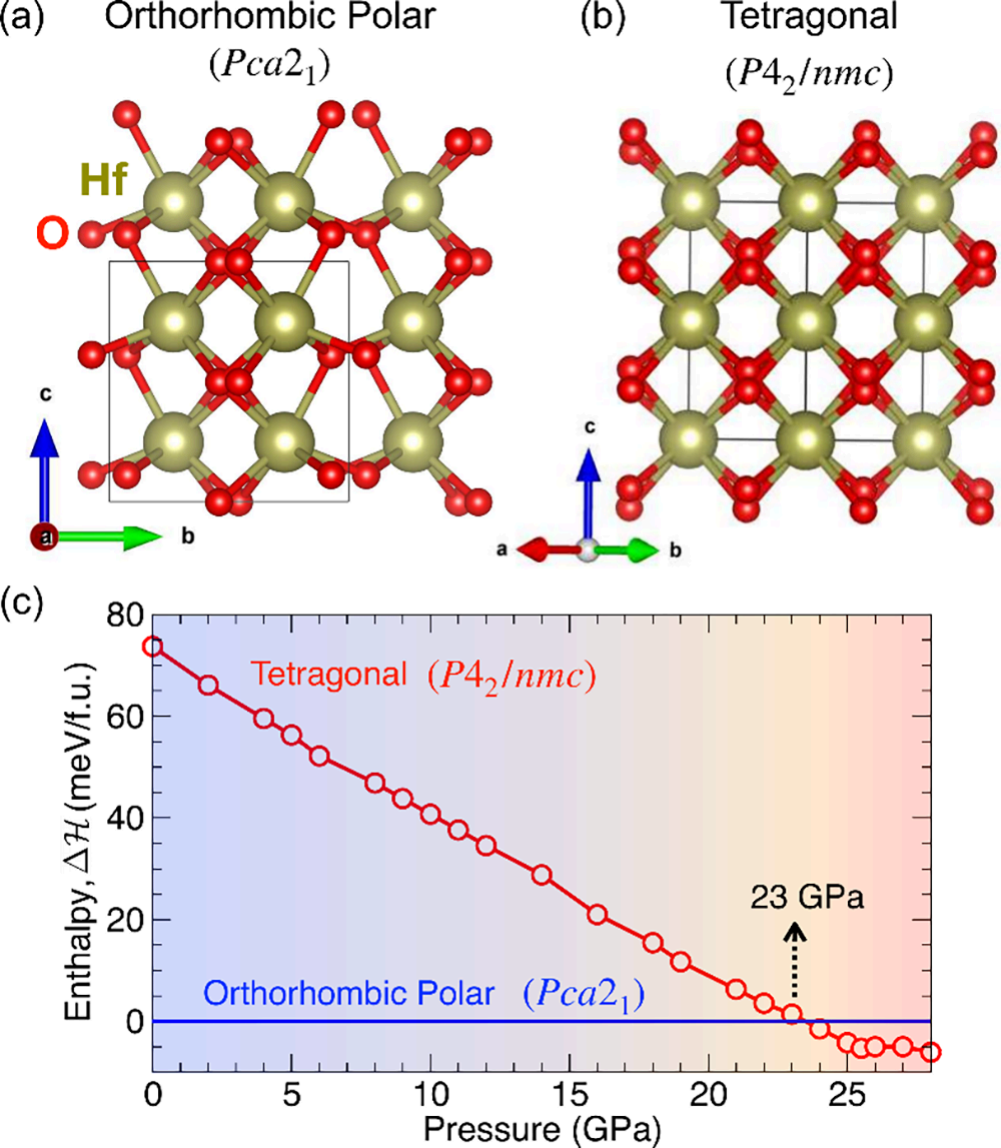
(a,b) Crystal structures of the orthorhombic polar (*Pca*2_1_) and tetragonal (*P*4_2_/*mnc*) phases of hafnia. The pressure-driven reaction rearranges the oxygen centers while the hafnium ions remain fixed. This type of subtle difference makes it challenging to correctly identify a phase using a single technique. (c) Calculated enthalpy difference () vs pressure for pure HfO_2_. The tetragonal phase is predicted to become enthalpically more favorable above 23 GPa.

In this work, we combined accelerator-based infrared absorption and Raman scattering with diamond anvil cell techniques to reveal the spectroscopic response of HfO_2_:12%Y under compression. Remarkably, pressure drives a reaction in which polar orthorhombic hafnia is transformed into the tetragonal *P*4_2_/*nmc* phase above 22 GPa [Figure 1]. We confirm the identify of this higher symmetry phase using previously predicted signature phonon modes of tetragonal hafnia^29,30^ and support the assignment with symmetry arguments and an analysis of the energy landscape. Frequency vs pressure trends also reveal a Raman-active *A*_1*g*_ symmetry phonon in the tetragonal phase with a negative mode Grüneisen parameter that has important consequences for the development of competing phases. These findings advance the understanding of presssure-driven transitions in binary oxides, establish the viability of tetragonal hafnia at room temperature in single crystal form, and suggest that extreme pressure conditions may be capable of tuning between other metastable phases of hafnia.^27,38−45^ The fundamental excitations of the lattice are also relevant to the ferroelectric switching pathway, phononic engineering, and heat management.^12,19,20^

## Methods

High quality hafnia crystals stabilized with yttrium (chemical formula HfO_2_:*x*Y, where *x* = 12%) were grown by laser-diode heated floating zone techniques with rapid cooling.^32^ A small, well-shaped piece of the polar orthorhombic material was loaded into a diamond anvil cell equipped with type IIas or ultralow fluorescence diamonds with 500 μm culets. We used a 75 μm gasket hole and either petroleum jelly or KBr as a pressure medium (depending on the measurement) to ensure quasi-hydrostatic pressure conditions. Fluorescence from an annealed ruby ball was used to determine pressure and to ensure that the sample remained under quasi-hydrostatic conditions at all pressures [Figure S1, Supporting Information].^46^ Care was taken to optimize optical density in order to reveal the features of interest. Taking advantage of the stable, high-brightness beam, synchrotron-based infrared spectroscopy (60–680 cm^–1^; 4 cm^–1^ resolution) was performed using a Bruker Vertex 80 equipped with a bolometer detector at the 22-IR-1 beamline at the National Synchrotron Light Source II at Brookhaven National Laboratory. Raman scattering was carried out with a 532 nm laser (≤1 mW), backscattering geometry, a 1800 line/mm grating, and a liquid N_2_-cooled CCD detector. Pressure was increased stepwise between 0 and approximately 27 GPa. Sample fluorescence increased significantly above this pressure, so we did not go further. The structural phase transition is reversible upon release of pressure after about 20 min [Figure S2, Supporting Information]. Complementary first-principles density functional theory (DFT) calculations were performed using the Vienna Ab initio Simulation Package (VASP)^47−49^ within the projector-augmented wave framework.^50^ The generalized-gradient approximation was employed to calculate the exchange-correlation functional.^51^ Details are available in Supporting Information.^52^

## Results and Discussion

### Creating Tetragonal Hafnia under Compression at Room Temperature

**Figure 2:**
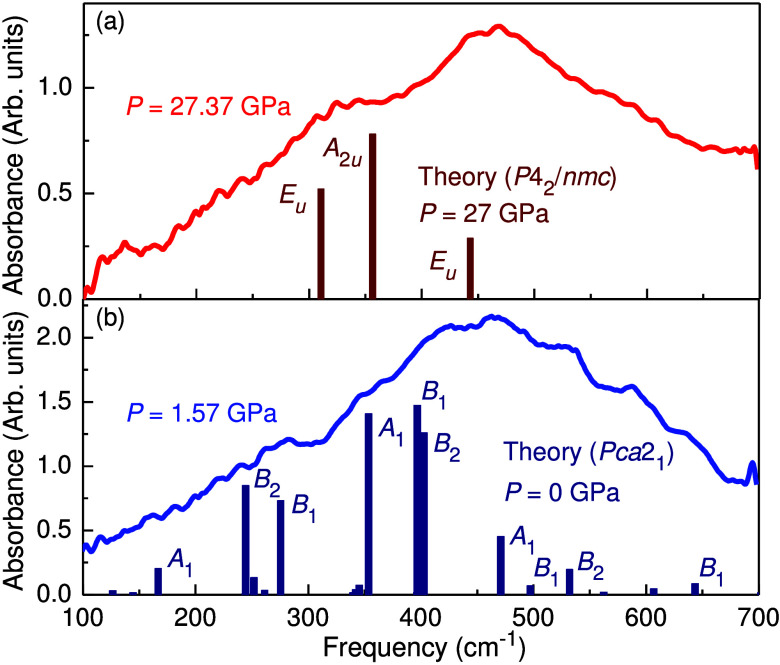
Infrared absorption of HfO_2_:12%Y in the polar orthorhombic (blue) and nonpolar tetragonal (red) phases at room temperature. Theoretically predicted mode frequencies and intensities are included for comparison. Select mode symmetries are labeled, and space groups are indicated.

Figure 2 displays the infrared absorbance of HfO_2_:12%Y. The ambient pressure phase of this system is already established as polar orthorhombic hafnia (*Pca*2_1_)^32^ with signature *A*_1_ symmetry modes at 167 and 471 cm^–1^ and *B*_1_ modes at 497 and 643 cm^–1^.^29^ The spectrum of the tetragonal phase material (*P*4_2_/*nmc*) is fully formed and no longer changing by 26 GPa with signature *E*_*u*_ symmetry modes at 305 and 445 cm^–1^ and an *A*_2*u*_ mode at 355 cm^–1^. The theoretically predicted vibrational fingerprints of this phase are consistent with the measured result, thus confirming the identity of the high pressure phase. That said, the features are significantly broadened due to Y substitution. Even so, the results demonstrate the power of vibrational spectroscopy for phase identification. As a reminder, this approach is successful is because the irreducible representations of distinct crystal phases exhibit the full crystal symmetry.^29,36^ The clear phonons in the high pressure phase also suggest that tetragonal HfO_2_:12%Y is crystalline. This supposition is further confirmed by the fact that the reaction of polar orthorhombic hafnia to create tetragonal hafnia is reversible upon release of pressure [Supporting Information].

**Figure 3:**
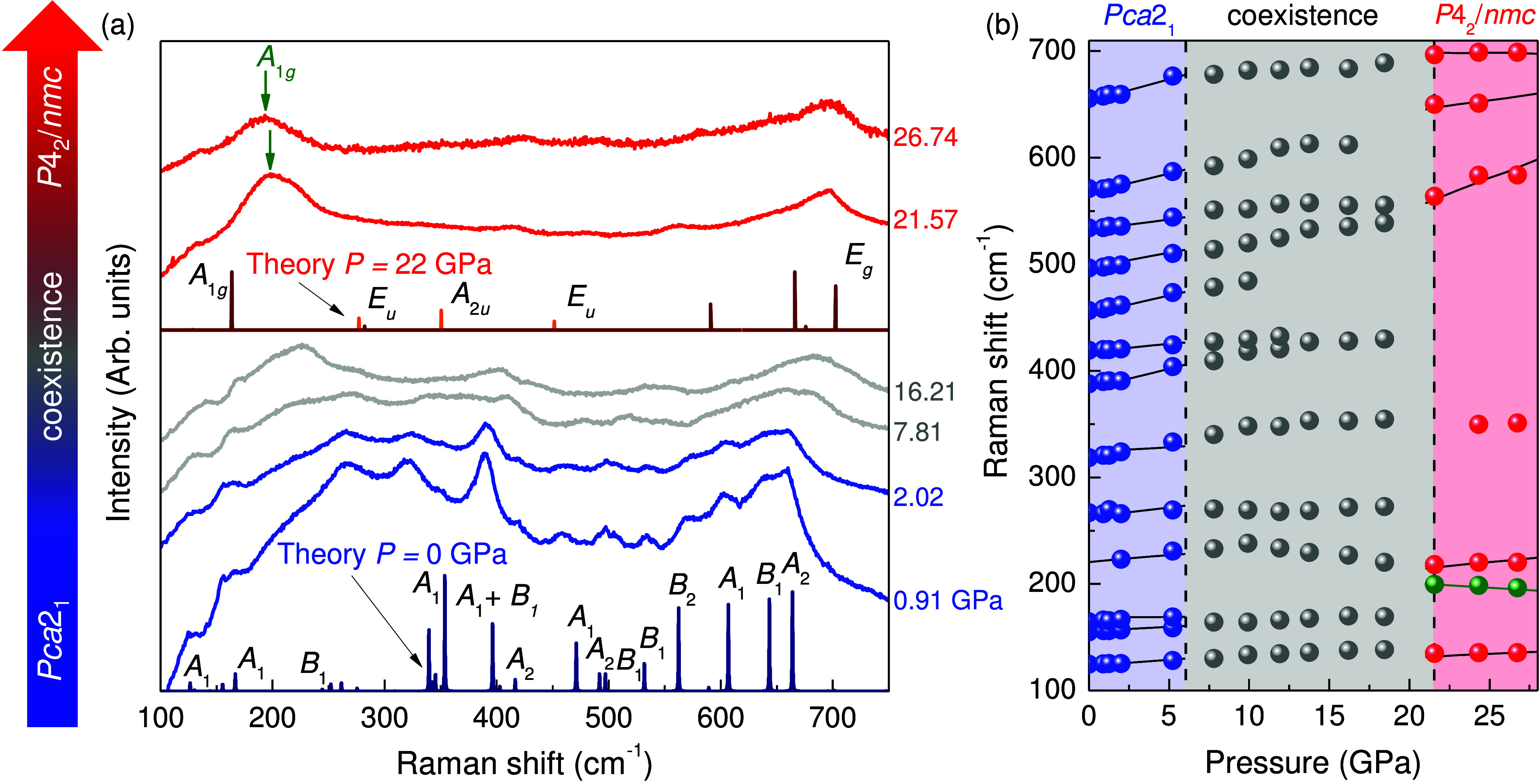
(a) Raman scattering response of HfO_2_:12%Y as a function of pressure at room temperature. Compression drives the polar orthorhombic form (blue) into a wide mixed-phase region (gray) and subsequently into the nonpolar tetragonal phase (red). The calculated mode patterns are shown for comparison, and symmetries are labeled accordingly. Y-induced features are shown in orange for the tetragonal phase. The space groups and coexistence range are indicated. (b) Frequency vs pressure for HfO_2_:12%Y at 300 K. An *A*_1*g*_ symmetry phonon near 200 cm^–1^ displays a negative mode Grüneisen parameter in the tetragonal phase (green spheres).

Figure 3 displays the Raman scattering response of HfO_2_:12%Y as a function of pressure at 300 K. Here, we can see the systematic progression from polar orthorhombic to mixed to tetragonal phase hafnia. The signature Raman-active modes of polar orthorhombic hafnia include *A*_1_ symmetry features at 354, 396, and 471 cm^–1^ and *B*_2_ symmetry structures at 532 and 562 cm^–1^ at ambient conditions.^29^ The Raman-active modes in our sample are broader that what one might encounter in an epitaxial thin film. This is attributable to the Y substitution which is required to stabilize the polar orthorhombic phase in HfO_2_:12%Y. These vibrational modes shift only slightly under pressure—as expected for a hard material. The two-phase region between 8 and 20 GPa might be a sign of nonhydrostaticity, but the fact that a second crystalline phase with sharp, clear phonons emerges at still higher pressures argues against such an interpretation and instead suggests that it is merely an extended transition region. Disorder is also different than the development of a distinct structural phase in that spectral features would be expected to continue diminishing and broadening—which is not the case. Tetragonal hafnia begins to appear above 20 GPa, and it is well-established by 26 GPa. We did not go to significantly higher pressures because the sample fluoresces strongly under these conditions. Whether this is due to the luminescence of oxygen vacancies in HfO_2_^53^ or an intrinsic property of the high pressure phase is not known at this time. In any case, the measured Raman response of tetragonal hafnia under pressure compares exceptionally well with our calculations, with strong *A*_1*g*_ and *B*_1*g*_ symmetry modes predicted at 129 and 674 cm^–1^, respectively, along with two *E*_*g*_ symmetry modes at 614 and 703 cm^–1^ at 26 GPa. In addition to the fundamental Raman-active modes of tetragonal hafnia, a few extra ungerade symmetry features are activated by Y inclusion. This is because local Y impurities lead to static disorder which breaks local symmetries.^29^ Clearly polar orthorhombic hafnia is much more than a high-κ gate dielectric for CMOS technology.^54^ It is a gateway to stabilizing the more elusive phases of hafnia.

### Frequency vs Pressure Trends and Soft Mode in the Tetragonal Phase

**Figure 4:**
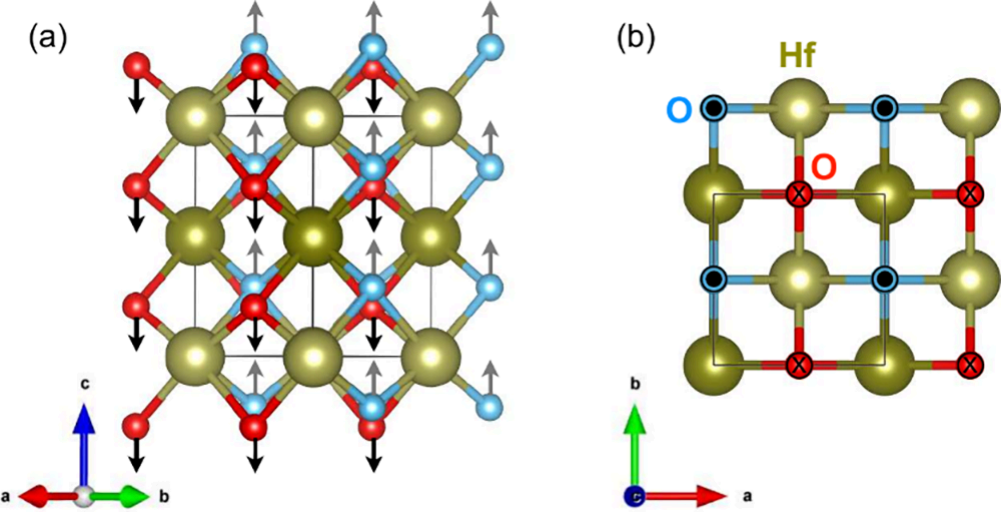
Displacement pattern of the Raman-active *A*_1*g*_ symmetry mode of tetragonal hafnia showing out-of-phase motion of the oxygen centers viewed in different orientations. Inequivalent oxygen atoms are shown in red and blue. This is the only phonon that softens in the tetragonal phase. All others stiffen. The *A*_1*g*_ mode in the tetragonal phase is analogous to the *X*_2_^–^ mode in cubic hafnia.

With the spectroscopic signatures across the structural phase transition on a firm foundation, we can compare frequency vs pressure trends with our lattice dynamics calculations. Overall, the Raman-active phonons harden under pressure [Figure 3(b)]. The *A*_1*g*_ symmetry mode near 200 cm^–1^ in the tetragonal phase is different. It softens under compression and in fact hosts a negative mode Grüneisen parameter [Table 1]. The experimental softening of the *A*_1*g*_ mode is on the order of 5 or 6 cm^–1^. This is actually a large red shift considering that the normal trend under pressure is to harden. Example peak fits are available in the Supporting Information, although the trend is apparent even with the naked eye. Hafnia has been predicted to host several different types of soft modes, although to our knowledge this is the first such mode-softening that has been observed in an experiment. Our calculations reveal that the soft *A*_1*g*_ phonon in the tetragonal phase bears a striking similarity to the unstable zone-boundary *X*_2_^–^ mode of cubic hafnia which is responsible for the cubic-to-tetragonal phase transition with decreasing temperature (2900 > *T* > 2073 K).^6,12,13,25^Figure 4 displays the displacement pattern of this vibrational mode.

**Table 1:** Summary of Mode Grüneisen parameters (γ_*i*_’s) for the Raman-Active Vibrational Features in the Tetragonal Phase of HfO_2_:12%Y^a^

ω_*i*_ (cm^–1^)	mode symmetry	*∂ω_i_*/*∂P* (cm^–1^/GPa)	γ_*i*_
134.7	*E*_*g*_	0.11	0.20
**199.6**	*A*_1*g*_	**-0.66**	**-0.81**
218.1	*B*_1*g*_	0.43	0.48
349.7	*A*_2*u*_	0.46	0.32
563.5	*E*_*g*_	4.0	1.73
650.2	*B*_1*g*_	0.36	0.14
696.7	*E*_*g*_	0.39	0.14

a The ω_*i*_’s were obtained at 21 GPa, and the *∂ω*/*∂P*’s were extracted from our measurements between 21 and 27 GPa.

In pristine HfO_2_, the rate of *A*_1*g*_ mode softening is predicted to increase drastically above 20 GPa [Figure S3, Supporting Information], revealing the crucial role of this particular phonon mode in the phase transformation process. It is worth noting that, as compared to our theoretical calculations for pure tetragonal HfO_2_, the experimental data shows relatively smaller mode softening [Figure 3(b)]. We attribute the difference to the presence of Y substitution in the HfO_2_:12%Y crystals. However, both in theory and experiment, the *A*_1*g*_ phonon stands out as the sole feature with a negative mode Grüneisen parameter among all other Raman-active modes in the tetragonal phase. Observation of the *A*_1*g*_ soft mode in tetragonal hafnia suggests that this phonon drives the phase transition toward the cubic (*Fm*3̅*m*) phase, as predicted theoretically.^6,12,13,25^ The cubic phase is predicted to form at higher pressures above 28 GPa^36^ and has been stabilized as HfO_2_:20%Y by laser floating zone techniques.^29,32^ We have not been able to detect its presence in our experiments due to strong fluorescence interference.

### The Intermediate Pressure Coexistence Regime

Another remarkable aspect of HfO_2_:12%Y under pressure is the unusually wide mixed-phase region between approximately 8 and 20 GPa where polar orthorhombic (*Pca*2_1_) and nonpolar tetragonal (*P*4_2_/*nmc*) phases coexist [Figure 3]. Similar phenomena are often observed in other parameter spaces. For instance, Schroeder et al. recently discovered a wide coexistence region in Hf_*x*_Zr_1–*x*_O_2_, although in this family of mixed metal oxides, the polar orthorhombic to nonpolar tetragonal transition develops due to changes in doping and oxygen stoichiometry.^26^ In our case, we can see that the peaks associated with the polar orthorhombic phase diminish in intensity whereas those associated with the tetragonal phase start to grow, demonstrating that what replaces the orthorhombic phase is indeed the tetragonal phase. The properties of these coexistence regimes are highly under-explored. The spectra in this pressure range are also noticeably broader than those in the *Pca*2_1_ and *P*4_2_/*nmc* states due to (i) the complex and overlapping mode patterns in the mixed phase and (ii) the fact that we are getting nearer to resonance with the exciting laser under compression. In any case, this system provides the opportunity to study the conditions of coexistence, tune the relative amounts of each phase by adjusting pressure, and even distinguish the relative contribution of the different phases by their phonon signatures—even though the two phases have similar structures. A phase change over such a wide range of pressures may host interesting piezoelectric effects.^26^

### Evaluating Phonon Lifetimes

The signature modes in the high pressure tetragonal phase of HfO_2_:12%Y are noticeably broader than those in the low pressure polar orthorhombic form suggesting important differences in phonon lifetimes. The latter is a useful quantity related to the Heisenberg uncertainty principle that can be calculated as τ = *ℏ*/γ where γ is the phonon line width.^55^ Typical phonon lifetimes for the orthorhombic polar phase are between 1 and 1.8 ps, whereas the phonon lifetimes are much shorter in the tetragonal phase (0.05–0.07 ps). The former are similar to Si (1.6–2 ps)^56^ whereas the latter are comparable to semiconductors like 1T-HfS_2_ (0.03–0.4 ps).^57^ Employing a characteristic phonon velocity of 4000 m/s, we find mean free paths of between 4000 and 7200 pm in the polar orthorhombic phase. The mean free path is shorter in the tetragonal phase due to an increased number of scattering events.

### Phase Competition in This System

It is of great interest to understand the energetics and symmetry rules of this rearrangement. Figure 1(c) displays the enthalpy vs pressure curve for this system calculated from first principles. Polar orthorhombic hafnia is the reactant at ambient conditions, so it starts as the ground state; the tetragonal phase is at much higher energy. With increasing pressure, the energy of the tetragonal form decreases systematically, approaching that of polar orthorhombic hafnia. The two enthalpy surfaces cross near 23 GPa, in excellent agreement with our measurements. Above this pressure, the ground state is tetragonal—different than what might be expected based upon the phase diagram of ref (27). We attribute this result to the different starting point (pure monoclinic HfO_2_ vs polar orthorhombic HfO_2_:12%Y). As shown by the energy axis, 23 GPa is equivalent to 75 meV/f.u. Compression to more extreme conditions (beyond 30 GPa) is predicted to stabilize the cubic phase.^36^

## Conclusion

To summarize, we measured the vibrational properties of polar orthorhombic hafnia under extreme conditions and uncover a compression-driven reaction that drives to the tetragonal (*P*4_2_/*nmc*) form above 22 GPa. This transformation, which takes place at room temperature, reveals that the tetragonal phase can be present at 300 K if the compression processes is begun with the *Pca*2_1_ form of hafnia (in this case, HfO_2_:12%Y) rather than a monoclinic crystal. Analysis of the energy landscape confirms this local minimum and further suggests that the cubic phase should be accessible above 30 GPa. Observation of an *A*_1*g*_ symmetry mode with negative mode Grüneisen parameter in the tetragonal phase is in line with these predictions. These findings raise the possibility that other forms of hafnia might be accessed using similar high pressure methods, at least partially obviating the current ultrahigh temperature growth conditions and moving a step closer to realizing the full potential of scale-free ferroelectricity in useful devices. The ability to identify tetragonal hafnia may also support greater understanding of the switching pathway in the polar material.

## Supporting Information Available

Technical details regarding hydrostaticity in our high-pressure measurements, theoretical calculations, analysis of mode Grüneisen parameters, obtained mode Grüneisen parameters for the tetragonal and orthorhombic-polar phases of HfO_2_, peak fitting, crystal growth, and pressure-dependence of zone-center phonon frequencies for the tetragonal phase of hafnia (PDF)
