# Supplementary material for: Pressure-Driven Polar Orthorhombic to Tetragonal Phase Transition in Hafnia at Room Temperature
Source: Chem Mater. 2025 Feb 17;37(5):1820–5. doi: 10.1021/acs.chemmater.4c02596 (PMC11905205; doi:10.1021/acs.chemmater.4c02596)
Supplement: Supplementary file 1 — cm4c02596_si_001.pdf [file cm4c02596_si_001.pdf]

Supporting Information for "Pressure-Driven Polar Orthorhombic to Tetragonal Phase Transition in Hafnia at Room Temperature"

Janice L. Musfeldt,[1,2,*] Sobhit Singh,[3,4] Kevin A. Smith,[1] Xianghan Xu,[5,6]
Sang-Wook Cheong,[5,6] Zhenxian Liu,[7] David Vanderbilt,[5] and Karin M. Rabe[5]

[1]*Department of Chemistry, University of Tennessee, Knoxville, Tennessee 37996, USA*

[2]*Department of Physics and Astronomy, University of Tennessee, Knoxville, Tennessee 37996, USA*

[3]*Department of Mechanical Engineering, University of Rochester, Rochester, New York 14627, USA*

[4]*Materials Science Program, University of Rochester, Rochester, New York 14627, USA*

[5]*Department of Physics and Astronomy, Rutgers University, Piscataway, New Jersey 08854, USA*

[6]*Rutgers Center for Emergent Materials, Rutgers University, Piscataway, New Jersey 08854, USA*

[7]*Department of Physics, University of Illinois Chicago, Illinois 60607-7059, USA*

[*] musfeldt@utk.edu

Supplementary Note 1: Maintaining quasi-static conditions inside the cell 3

Supplementary Note 2: Reversibility on pressure cycling 3

Supplementary Note 3: Example peak fits in the tetragonal phase 4

Supplementary Note 4: Analyzing the mode Grüneisen parameters 4

Supplementary Note 5: Tetragonal phase phonons frequencies under pressure 6

Supplementary Note 6: Details of our calculations 7

Supplementary Note 7: Crystal growth details 7

References 8

Supplementary Note 1: Maintaining quasi-static conditions inside the cell

Figure S1(a) displays the fluorescence of the annealed ruby ball as a function of pressure inside the diamond anvil cell. The $R1$ and $R2$ peaks are well separated and fairly symmetric with increasing pressure, indicating that the sample is in a quasi-hydrostatic environment. Based upon the shape of the fluorescence response over the full pressure range shown here [Fig. S1] as well as the phonon lineshapes of hafnia, we estimate that the deviation from a perfect hydrostatic pressure environment is on the order of a few percent.

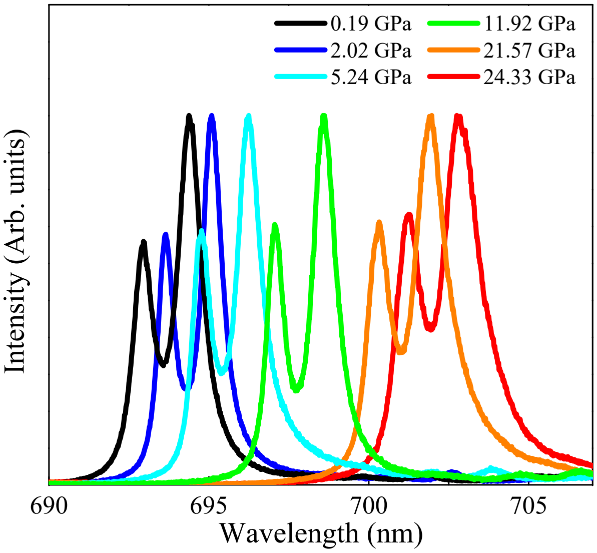

FIG. S1. Typical ruby fluorescence spectra at selected pressures. Note the clear separation of the $R1$ and $R2$ peaks and the lack of asymmetry indicating that a quasi-hydrostatic environment is maintained in the diamond anvil cell. These data were taken at room temperature and indicate quasi-hydrostatic conditions over the full pressure range.

The choice of pressure media also influences hydrostaticity. In principle, only a sample surrounded by a liquid pressure medium should be considered to exist in perfect hydrostatic conditions. That said, KBr, and petroleum jelly are common pressure transmitting media, and although they do not generate true hydrostatic conditions, they generate quasi-hydrostatic consitions for the samples that we studied because all of the different phases of hafnia are much harder than our chosen pressure media. As a result, the non-hydrostatic effects on the samples can be neglected.

Supplementary Note 2: Reversibility on pressure cycling

Figure S2 displays the vibrational response of HfO_2:12%Y both before and after pressure cycling to 26 GPa. The results are identical indicating that the tetragonal phase is not metastable upon pressure release.

FIG. S2. Comparison of HfO_2:12%Y before (ambient) and after (release) compression. Both Raman scattering spectra are in reasonable agreement, indicating that the tetragonal phase is not metastable after pressure release.

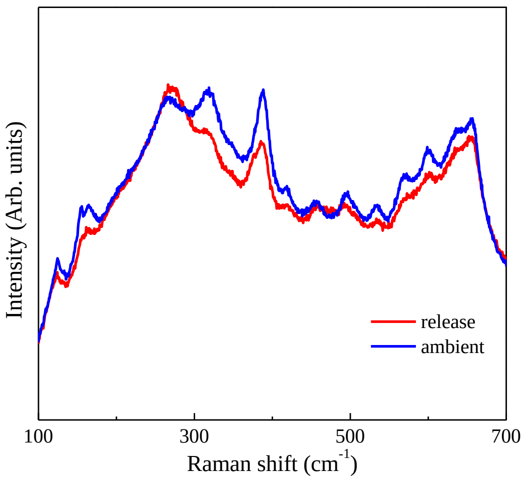

Supplementary Note 3: Example peak fits in the tetragonal phase

Figure S3 shows two example peak fits that focus on the softening of the A_{1g} mode in the tetragonal phase. This trend is readily apparent by eye as well. Notice that in addition to the primary contribution of the A_{1g} symmetry mode of tetragonal hafnia, we also take account of Y incorporation. The latter activates several additional small features.

FIG. S3. Model Voigt oscillator fitting of the hafnia (red) and yttria-related (blue) peaks in the Raman spectra at (a) 21.57 and (b) 26.74 GPa illustrating the softening of the A_{1g} mode in greater detail. The pink curve provides the simulated spectra by combining the two components, indicating the quality of the fit to the experimental data (black).

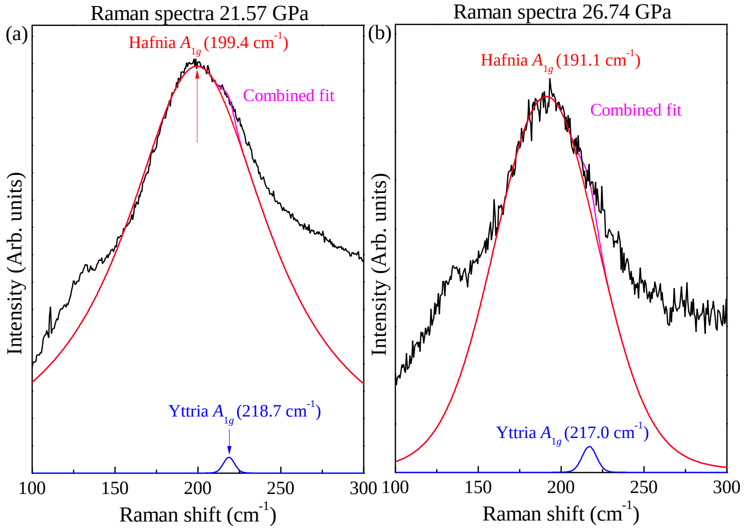

Supplementary Note 4: Analyzing the mode Grüneisen parameters

We calculate the mode Grüneisen parameters as:

$$\gamma_i = \frac{B}{\omega_i}\frac{\partial \omega_i}{\partial p}, \tag{1}$$

where B is the bulk modulus, ω_i is the frequency of a given mode, and $\frac{\partial \omega_i}{\partial p}$ is obtained by extracting the slope from plots of frequency vs. pressure in the relevant pressure range. We use the DFT calculated bulk modulus of 244.4 GPa to estimate the individual mode Grüneisen parameters. The coefficient of thermal expansion is related to the Grüneisen parameters as:

$$\alpha = \frac{\gamma_{av} c_v}{3B}, \tag{2}$$

where c_v is the molar heat capacity at constant volume and γ_{av} is the mean of all individual mode Grüneisen parameters.[1,2] A negative Grüneisen parameter indicates a soft mode and the possibility of negative thermal expansion. However, given that γ_{av} remains positive, α will remain positive in this system.

TABLE S1. Calculated mode Grüneisen parameters for the Raman-active vibrational features in the orthorhombic polar phase of HfO_2:12%Y. The ω_i's were obtained at ambient conditions, and the $\partial\omega/\partial P$'s were extracted in the 0 - 6 GPa pressure range from our experimental data.

ω_i (cm^{-1})	mode symmetry	$\frac{\partial \omega_i}{\partial p}$ (cm^{-1}/GPa)	γ_i
124.3	A_1	0.72	1.42
155.2	A_2	0.66	1.04
164.8	A_1	0.80	1.19
218.2	A_1	2.38	2.67
266.5	B_2	0.51	0.46
318.0	A_2	2.86	2.20
386.5	B_2	3.15	1.99
418.7	A_2	1.02	0.59
455.7	A_1	3.32	1.78
495.2	B_1	2.70	1.33
532.9	B_1	2.03	0.93
568.8	B_2	3.33	1.43
653.8	B_1	4.14	1.55

TABLE S2. Calculated mode Grüneisen parameters for the Raman-active vibrational features in the tetragonal phase of HfO_2:12%Y. The ω_i's were obtained at 21 GPa, and the $\partial\omega/\partial P$'s were extracted in the 21 - 27 GPa range from our experimental measurements.

ω_i (cm^{-1})	mode symmetry	$\frac{\partial\omega_i}{\partial p}$ (cm^{-1}/GPa)	γ_i
134.7	E_g	0.11	0.20
199.6	A_{1g}	-0.66	**-0.81**
218.1	B_{1g}	0.43	0.48
349.7	A_{2u}	0.46	0.32
563.5	E_g	4.0	1.73
650.2	B_{1g}	0.36	0.14
696.7	E_g	0.39	0.14

Supplementary Note 5: Tetragonal phase phonons frequencies under pressure

The tetragonal unit cell contains a total of 6 atoms (*i.e.*, 2 f.u.), hence, there are 15 optical phonon modes having the following irreducible representations at the Brillouin zone center:

$$\Gamma_{tetra} = 1\,A_{1\text{g}} \oplus 2\,B_{1\text{g}} \oplus 3\,E_{\text{g}} \oplus 1\,A_{2\text{u}} \oplus 1\,B_{2\text{u}} \oplus 2\,E_{\text{u}}. \tag{3}$$

The $A_{2\text{u}}$ (or Γ_2^-) and E_{u} (or Γ_5^-) modes are infrared active, whereas the $A_{1\text{g}}$ (or Γ_1^+), $B_{1\text{g}}$ (or Γ_3^+), and E_{g} (or Γ_5^+) modes are Raman active. The $B_{2\text{u}}$ (or Γ_4^-) hyper-Raman active mode is silent in typical Raman and infrared experiments. The pressure dependence of these optical modes, calculated using the density-functional perturbation theory, is shown in Fig. S4. Our calculations reveal that only the Raman-active A_{1g} mode softens with increasing pressure. This mode is shown in magenta. The rate of pressure-induced softening of the A_{1g} mode drastically increases above 20 GPa suggesting the occurrence of a pressure-driven phase transition. Indeed, our enthalpy calculations predict that the cubic phase (SPG#225) becomes enthalpically more favorable compared to the tetragonal phase beyond 30 GPa pressure. All other Raman-active modes of the tetragonal phase harden with increasing pressure, thus exhibiting positive mode Grüneisen parameters. This is in excellent agreement with our experimental observations as shown in Table S2. We emphasize that these curves model the response of tetragonal hafnia over the full pressure range in the absence of Y substitu-

tion. In fact, Y substitution dampens the softening in the real material compared with the model, and the tetragonal phase is only observed in the experiment above 20 or 22 GPa. The calculations give a much larger red shift than experimentally observed because they are not encumbered with Y.

Supplementary Note 6: Details of our calculations

All first-principles density functional theory (DFT) calculations were performed using the Vienna Ab initio Simulation Package (VASP)[3–5] within the projector-augmented wave framework.[6] The generalized-gradient approximation as parameterized by Perdew, Burke, and Ernzerhof for solids (PBEsol) was employed to calculate the exchange-correlation functional.[7] The total energy convergence and residual force convergence criteria were set to 10^{-7} eV and 10^{-3} eV/ Å, respectively. For numerical sampling of the reciprocal space, we used a Monkhorst-Pack k-mesh[8] of size 12×12×8 for tetragonal phase and 8×8×8 for the orthorhombic polar phase. The cutoff energies for the plane-wave basis set was set at 600 eV. DFT optimized lattice parameters at 0 GPa are $a = b = 3.548$, $c = 5.102$ Å for the tetragonal phase ($P4_2/nmc$) and $a = 5.203$, $b = 5.002$, $c = 5.018$ Å for the orthorhombic polar phase ($\mathrm{Pca2_1}$). Phonon frequencies were calculated using the density-functional perturbation theory. The Bilbao Crystallographic Server[9] and PHONOPY package[10] were utilized to determine the symmetry of the phonon eigenvectors. MechElastic package was utilized to calculate the elastic properties and bulk modulus.

Supplementary Note 7: Crystal growth details

The HfO_2:12%Y single crystal used in this study was grown utilizing a laser floating zone technique.[12] High-purity powder of Y_2O_3 (900°C overnight baked) and HfO_2 in molar ratio Y:Hf = 0.12:0.88 were mixed and sintered at 1600°C for 20 hours with one intermediate grinding. The product was shaped into a rod and sintered at 1600°C again. The growth was performed in air flow and a rate of 5 mm per hour. Then the laser power was reduced to slightly below the melting point and scanned through the crystal rod at a fast rate of 300 mm per hour to stabilize the meta-stable polar orthorhombic phase. The phase purity and $Pca2_1$ crystal structure of the final product were confirmed using x-ray diffraction and transmission electron diffraction.[12] Ferroelectricity was examined by a polarization versus

FIG. S4. DFT calculated zone-center phonon frequencies (ω_Γ) vs. pressure for tetragonal ($P4_2/mnc$) hafnia. Polar phonon modes are shown using dashed lines. Only one A_{1g} mode (magenta) exhibits a negative Grüneisen parameter, whereas all other phonons in the tetragonal phase harden with increasing pressure. The rate of pressure-induced softening of the A_{1g} mode drastically increases above 20 GPa.

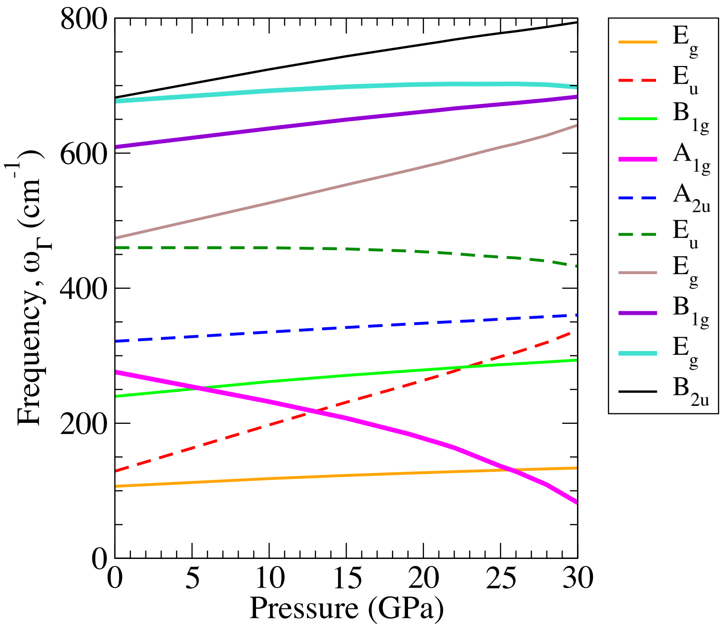

electric field hysteresis measurement performed in a Radiant ferroelectric test system.[12]

[S1] Ritz, E. T. and Benedek, N. A. Interplay between phonons and anisotropic elasticity drives negative thermal expansion in $PbTiO_3$. *Phys. Rev. Lett.* **121**, 255901 (2018).

[S2] Ashcroft, N. W. and Merman, N. D. *Solid State Physics.* D. G. Crane, ed. (Saunders College, Philadelphia 1976). p. 493.

[S3] Kresse, G. and Furthmüller, J.Efficient iterative schemes for *ab initio* total-energy calculations using a plane-wave basis set. *Phys. Rev. B* **54**, 11169–11186 (1996).

[S4] Kresse, G. and Furthmüller, J. Efficiency of $ab - initio$ total energy calculations for metals and semiconductors using a plane-wave basis set. *Comput. Mater. Sci.* **6**, 15-50 (1996).

[S5] Kresse, G. and Joubert, D. From ultrasoft pseudopotentials to the projector augmented-wave method. *Phys. Rev. B* **59**, 1758-1775 (1999).

[S6] Blöchl, P. E. Projector augmented-wave method. *Phys. Rev. B* **50**, 17953-17979 (1994).

[S7] Perdew, J. P. *et al.*, Restoring the density-gradient expansion for exchange in solids and surfaces. *Phys. Rev. Lett.* **100**, 136406 (2008).

[S8] Monkhorst, H. J. and Pack, J. D. Special points for Brillouin-zone integrations. *Phys. Rev. B* **13**, 5188–5192 (1976).

[S9] Kroumova, E. *et al.* Bilbao crystallographic server: useful databases and tools for phase-transition studies. *Phase Transit.* **76**, 155–170 (2003).

[S10] Togo, A. and Tanaka, I. First principles phonon calculations in materials science. *Scr. Mater.* **108**, 1–5 (2015).

[S11] Singh, S. *et al.*, MechElastic: A Python library for analysis of mechanical and elastic properties of bulk and 2D materials. *Computer Physics Communications* **267**, 108068 (2021).

[S12] Xu, X. *et. al.*, Kinetically stabilized ferroelectricity in bulk single-crystalline HfO_2:Y. *Nature Mater.* **20**, 826-832 (2021).
